# Successful Extubation Using Heliox BiPAP in Two Patients with Postextubation Stridor

**DOI:** 10.1155/2017/1253280

**Published:** 2017-03-08

**Authors:** Pragya Punj, Premkumar Nattanmai, Pravin George, Christopher R. Newey

**Affiliations:** ^1^Department of Neurology, University of Missouri, 5 Hospital Drive, CE 540, Columbia, MO 65211, USA; ^2^Cleveland Clinic, Cerebrovascular Center, Neurological Institute, 9500 Euclid Avenue, Cleveland, OH 44195-5245, USA

## Abstract

Postextubation stridor is associated with significant morbidity. It commonly results in extubation failure after established medical treatment fails, such as nebulized epinephrine and/or intravenous steroids. The role of heliox (i.e., combination of helium and oxygen) in managing patients with postextubation stridor has not been fully established. We report two cases of postextubation stridor successfully treated with heliox delivered with bilevel positive airway pressure (BiPAP) after failure of standard medical therapy.

## 1. Introduction

Stridor is a common complication seen in 2 to 16% of patients after extubation [1]. It is generally short-term and self-relieving [1]. In severe cases, it can result in respiratory failure and prompt need to reintubate [1]. A cuff leak test can be used prior to extubation as a crude measure of predicting postextubation stridor [1]. The treatment of postextubation stridor typically consists of intravenous or nebulized corticosteroids combined with nebulized epinephrine [1]. When these medical therapies fail, reintubation is usually imminent [1]. Algorithms have been proposed, which include the use of inhaled helium/oxygen mixture, to treat postextubation stridor [1]. We describe here two cases of postextubation stridor who failed medical therapy and were successfully managed with the combination of 80% helium and 20% oxygen (i.e., heliox) delivered with bilevel positive airway pressure (BiPAP).

## 2. Cases

### 2.1. Case 1

A young Caucasian woman with history of anxiety arrived to the emergency department (ED) after a restrained motor vehicle accident. Her Glasgow Coma Scale (GCS) was 6. She was intubated for airway protection. Her computed tomography (CT) head showed right frontotemporal subdural hematoma and underlying severe contusion, parafalcine and tentorial subdural hematoma, right petrous temporal bone skull fracture, occipital skull fracture, and bilateral cavernous internal carotid artery dissections.

She improved neurologically over the next nine days and was ultimately extubated. However, she developed significant stridor requiring frequent racemic epinephrine nebulization and scheduled intravenous dexamethasone. Her work of breathing increased, and she was reintubated. She continued dexamethasone for 24 hours and was extubated 48 hours after reintubation. She again developed stridor with severe desaturation requiring reintubation. She was extubated 24 hours later to heliox (80% helium/20% oxygen) delivered with BiPAP for postextubation stridor. She remained on the heliox BiPAP for 24 hours and did not require reintubation.

### 2.2. Case 2

An elderly Caucasian woman with history of hypertension was brought to ED for left hemiparesis. She quickly worsened in the ED requiring emergent intubation for airway protection. Her head CT showed right temporal intracerebral hemorrhage (30 cc) with mass effect on the right lateral ventricle and a 5 mm right to left midline shift at the septum pellucidum. Over the next five days she improved neurologically and was extubated. After extubation, she had significant stridor that did not respond to racemic epinephrine nebulization or BiPAP ventilation. She desaturated and was in acute respiratory distress requiring reintubation. Intravenous dexamethasone was given for 48 hours. She was again extubated. Stridor, however, recurred. She was then placed on BiPAP with heliox (80% helium/20% oxygen) for 6 hours without need for reintubation.

## 3. Discussion

We presented two illustrative cases of postextubation stridor in which usual treatment failed but responded to heliox combined with BiPAP therapy. Heliox is used in treatment of acute exacerbation of chronic obstructive pulmonary disease and asthma as well as in treatment of upper respiratory tract obstruction [2, 3]. Its utility in postextubation stridor has not been established.

Heliox is a combination of helium and oxygen. It was first described in the 1930s [2–4]. Helium is a biologically inactive gas with properties of low density and specific gravity [2]. Since density is inversely proportional to flow rate of a gas, the low density of heliox improves flow rate and reduces the resistance to flow in the airway, leading to decreased work of breathing [2, 3].

Heliox converts turbulent flow into laminar flow [2–4]. Flow is determined by Reynolds number [2]. As the Reynolds number increases, the turbulence of the flow will increase [2]. Importantly, Reynolds number declines with decrease in density and flow becomes more laminar in nature which increases gaseous exchange and oxygen reaching the tissues [2, 3].

In our cases, postextubation stridor was likely from laryngeal edema. Laryngeal edema typically occurs from mucosal damage resulting in ongoing inflammation and ultimately airway narrowing [1]. Identifying patients who may develop postextubation stridor is challenging [5]. Unfortunately, the endotracheal tube cuff leak test has been shown to be an inaccurate predictor of laryngeal edema [5]. In patients who develop postextubation stridor, the primary aim of treatment is to ensure adequate airflow, maintain patency of airway, and provide adequate oxygen delivery to tissues [1]. This may be accomplished by humidified oxygen therapy, nebulized racemic epinephrine, noninvasive positive pressure ventilation, or reintubation [5]. Another option is heliox. Heliox combined with BiPAP in our patients provided interim relief of postextubation stridor which had not responded to steroids, nebulized epinephrine, and/or BiPAP alone.

In conclusion, heliox combined with BiPAP is an option in managing postextubation stridor when standard medical therapy fails.
